# Mind your prevalence!

**DOI:** 10.1186/s13321-024-00837-w

**Published:** 2024-04-15

**Authors:** Sébastien J. J. Guesné, Thierry Hanser, Stéphane Werner, Samuel Boobier, Shaylyn Scott

**Affiliations:** Lhasa Limited, Granary Wharf House, 2 Canal Wharf, Leeds, West Yorkshire LS11 5PS UK

**Keywords:** Prevalence, Prevalence shift, Imbalanced, Balanced Matthews’ correlation coefficient, Calibrated metrics, Balanced metrics, Model validation, QSAR, Classification models

## Abstract

**Abstract:**

Multiple metrics are used when assessing and validating the performance of quantitative structure–activity relationship (QSAR) models. In the case of binary classification, balanced accuracy is a metric to assess the global performance of such models. In contrast to accuracy, balanced accuracy does not depend on the respective prevalence of the two categories in the test set that is used to validate a QSAR classifier. As such, balanced accuracy is used to overcome the effect of imbalanced test sets on the model’s perceived accuracy. Matthews' correlation coefficient (MCC), an alternative global performance metric, is also known to mitigate the imbalance of the test set. However, in contrast to the balanced accuracy, MCC remains dependent on the respective prevalence of the predicted categories. For simplicity, the rest of this work is based on the positive prevalence. The MCC value may be underestimated at high or extremely low positive prevalence. It contributes to more challenging comparisons between experiments using test sets with different positive prevalences and may lead to incorrect interpretations. The concept of balanced metrics beyond balanced accuracy is, to the best of our knowledge, not yet described in the cheminformatic literature. Therefore, after describing the relevant literature, this manuscript will first formally define a confusion matrix, sensitivity and specificity and then present, with synthetic data, the danger of comparing performance metrics under nonconstant prevalence. Second, it will demonstrate that balanced accuracy is the performance metric accuracy calibrated to a test set with a positive prevalence of 50% (i.e., balanced test set). This concept of balanced accuracy will then be extended to the MCC after showing its dependency on the positive prevalence. Applying the same concept to any other performance metric and widening it to the concept of calibrated metrics will then be briefly discussed. We will show that, like balanced accuracy, any balanced performance metric may be expressed as a function of the well-known values of sensitivity and specificity. Finally, a tale of two MCCs will exemplify the use of this concept of balanced MCC versus MCC with four use cases using synthetic data.

**Scientific contribution:**

This work provides a formal, unified framework for understanding prevalence dependence in model validation metrics, deriving balanced metric expressions beyond balanced accuracy, and demonstrating their practical utility for common use cases. In contrast to prior literature, it introduces the derived confusion matrix to express metrics as functions of sensitivity, specificity and prevalence without needing additional coefficients. The manuscript extends the concept of balanced metrics to Matthews' correlation coefficient and other widely used performance indicators, enabling robust comparisons under prevalence shifts.

**Supplementary Information:**

The online version contains supplementary material available at 10.1186/s13321-024-00837-w. It is also available at the GitHub repository MindYourPrevalence. The repository can be accessed at https://github.com/LhasaLimited/MindYourPrevalence.

## Introduction

Quantitative structure–activity relationship (QSAR) models play a crucial role in predicting and understanding the relationship between the chemical structure of a compound and its biological activity. These models use algorithms and statistical techniques to predict the activity or property of a compound based on its structural features. QSAR models have found wide applications in various fields, including drug discovery, toxicology, environmental sciences, and chemical risk assessment.

However, the perception of the reliability and accuracy of QSAR models heavily depends on the validation methodology. Model validation is a critical step in assessing the performance and generalizability of QSAR models before they can be confidently applied in real-world scenarios. The validation process and experimentation aim to determine the model's predictive power, its ability to generalise to unseen compounds, and its overall robustness [1, 2].

QSAR classification models are specifically designed to predict the class or category of a compound based on its structural features. They are commonly used to predict binary biological behaviour, such as toxicity (toxic/nontoxic), pharmacology (active/inactive), or other categorical endpoints. The internal validation for the development of QSAR classification models involves the construction of a confusion matrix from which statistical metrics may be computed to evaluate their performance. To evaluate the performance of a QSAR classification model, various statistical metrics may be calculated from a confusion matrix. These metrics include accuracy, sensitivity, specificity, positive and negative predictivity, Cohen’s kappa coefficient, F1 score, Matthews’ correlation coefficient, informedness (i.e., sum of sensitivity and specificity minus one) and markedness (i.e., sum of positive and negative predictivity values minus one). Accuracy measures the overall correctness of the predictions, while sensitivity and specificity indicate the model's ability to correctly identify positive and negative instances, respectively. Positive prediction evaluates the model's ability to correctly classify positive instances. External validation is also crucial for assessing the classifier's performance. In external validation, the classification model is tested on an independent dataset that was not used during the model development process. External validation ensures that the model can accurately predict the class of compounds beyond the dataset it was trained on and indicates its real-world applicability.

However, except for sensitivity and specificity, these performance metrics are dependent on the positive prevalence of datasets used during the validation study and where the positive prevalence quantifies the imbalance of the dataset with respect to positive instances. As a result, comparing performance metrics based on datasets with different positive prevalences may lead to incorrect conclusions.

Balanced accuracy, as well as sensitivity and specificity, is a well-known performance metric that naturally addresses this issue because it is independent of prevalence. Matthews’ correlation coefficient is also known to mitigate the issue around imbalanced datasets. However, in contrast to the balanced accuracy, this work will demonstrate that Matthews’ correlation coefficient is not fully immune to strong dataset imbalance and remains dependent on the positive prevalence. This dependency to positive prevalence can lead to misinterpretation of results when unbalanced metrics such as accuracy or Matthews’ correlation coefficient are compared under varying prevalence. Not accounting for prevalence effects may lead to incorrect model validations and erroneous conclusions. Greater awareness and adoption of calibrated/balanced metrics would mitigate these risks and improve the validity of model benchmarking in cases of nonconstant prevalence. Therefore, the main aims of this manuscript are threefold: to synthesise and harmonise previous work on prevalence dependence and balanced/calibrated metrics from across disciplines into a unified framework relevant for cheminformatics, provide new insights by deriving and extending the concept of balanced/calibrated metrics beyond balanced accuracy, and demonstrate the practical utility of balanced metrics for common use cases in cheminformatic model validation where prevalence shifts may occur.

## Related work

When evaluating a QSAR classification model, there are independent publications that discuss, study and/or propose a solution to the challenge of working under nonconstant or extreme positive prevalence in different scientific domains, such as machine learning, pattern recognition and screening tests [1, 3–9]. However, as far as we are aware, this is not described in the cheminformatic literature. Given the large number of performance metrics that may be calculated from a confusion matrix and the different methodologies that exist to tackle the problem, each publication discusses and studies the issue with various degrees of breadth, depth and perspective.

Luque et al*.* [3] propose an imbalance coefficient δ to quantify the degree of class imbalance in binary classification, analyse and categorise metrics by their imbalance bias, find geometric mean and informedness as most robust, and offer bias-corrected balanced metric versions to remove class imbalance effects when assessing classifier performance. Siblini et al*.* [4] propose calibrating precision-based metrics such as F1 and area under the precision-recall curve by reweighting true and false positives to match a reference positive class ratio, making the metrics more robust to class imbalance changes while retaining sensitivity to model performance changes, and recommend reporting the class ratio for proper interpretation. Brabec et al*.* [5] demonstrated that the positive predictivity and ranking of models by precision-based metrics depend directly on the class imbalance ratio, unlike the sensitivity and false positive rate, with mathematical adjustments preferred over subsampling to specified ratios; they argued for evaluation via plots spanning imbalance levels rather than individual metric values and mandatory reporting of class priors. Cooper II et al*.* [6] recommend using sensitivity and specificity as the primary measures to characterise the validity of carcinogen screening tests since positive predictivity also depends on carcinogen prevalence, emphasising the importance of reporting details on test substances and positivity criteria given the limitations of single summary indices. Heston et al*.* [7] criticise a study for incomplete reporting of negative predictivity of cardiac Magnetic Resonance Imaging without overall disease prevalence, which is needed for interpretation, and suggest reporting a standardised negative predictivity calculated at a fixed 50% prevalence along with the actual value to enable better comparison of diagnostic test performance intrinsically. Cayley et al. [1] argue that no single metric fully captures QSAR model performance for predicting mutagenicity, and proper expert interpretation of validation statistics requires considering factors such as chemical space, data balance, quality, model transparency, and suitability for decision support. Jeni et al. [8] find facial action unit detection metrics such as accuracy, F1, and Cohen’s kappa coefficient are attenuated under class imbalance, while the area under the receiving operating curve is robust, recommending that skew-normalised scores be reported alongside original metrics to enable unbiased comparisons across different datasets. Altman et al. [9] highlights that although positive and negative predictive values are vital for assessing real-world diagnostic performance, their interpretation requires careful consideration of the disease prevalence in the tested population, which substantially impacts the values. Although equations to calculate the positive and negative predictivity values for any prevalence, sensitivity and specificity are described, their origin or derivation is not. Other studies use mathematical expressions of the balanced positive and balanced negative predictivity values but do not explain or reference where they come from [10–12]. These mathematical expressions and the proof of their equivalence are described in Additional file 3.

The overarching theme across these prior publications is the need to carefully consider prevalence when evaluating classification models, as shifts or changes in prevalence can substantially impact certain performance metrics. This can lead to misinterpretation of validation results if the effect of prevalence is not accounted for when comparing metrics between datasets or experiments where prevalence differs. Solutions include the use of sensitivity and specificity, which are independent of the prevalence, and taking and reporting the value of the prevalence when looking at dependent performance metrics or balancing/calibrating them. The latter is the topic of this work.

## Definitions of the problem

### Definition

**Fig. 1:**
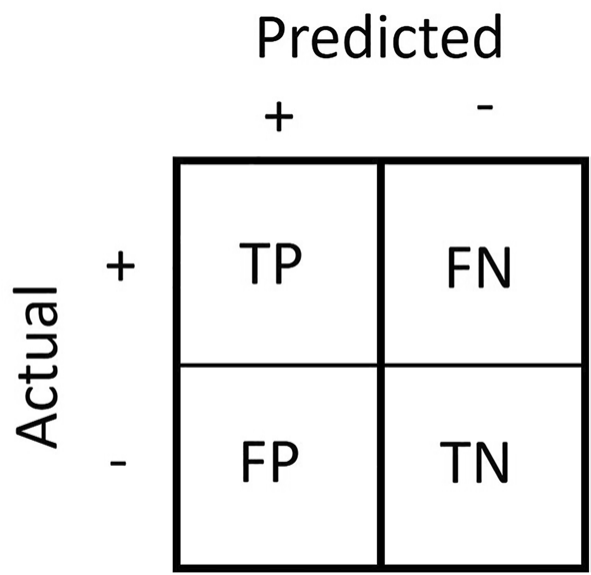
A confusion matrix summarises the counts of TP (true positive), FN (false positive), FP (false positive) and TN (true negative)

In the case of binary classification, a confusion matrix is a two-by-two table that summarises the performance of a QSAR classification model against a test set. The test set is made of *N* instances in total. The class of each instance is known and is either positive or negative. When a QSAR classification model is validated against a test set, four prediction types are possible: true positive, true negative, false positive and false negative. A prediction is a true positive (TP) when the model predicts a positive instance correctly and a false positive (FP) if the class of the instance is negative. A prediction is a true negative (TN) when the model predicts a negative instance correctly and a false negative (FN) if the class of the instance is positive. The count of these prediction types is tabulated where the rows are the actual class, and the columns are the predicted class, or vice versa. The resulting table is a confusion matrix, as illustrated in Fig. 1.

The total number of counts is *N*, the total number of instances in the test set. The confusion matrix has three degrees of freedom and may be used to compute a large range of statistics. Such statistics include positive prevalence, which is the relative frequency of positive instances within the test set as defined in Eq. (1). The relative frequency of true positives within the total number of positive instances of the test set is defined as the sensitivity in Eq. (2). The relative frequency of true negatives within the total number of negative instances of the test set is defined as the specificity in Eq. (3). Sensitivity and specificity may also be described as conditional probabilities but are out of the scope of this manuscript.

1 $$Pre=\frac{TP+FN}{N} \quad where \quad N \,= TP+FN+FP+TN$$

Equation 1. Positive prevalence definition from the prediction types tabulated in a confusion matrix.

2 $$Sen=\frac{TP}{TP+FN}$$

Equation 2. Sensitivity definition from the prediction types tabulated in a confusion matrix.

3 $$Spe=\frac{TN}{FP+TN}$$

Equation 3. Specificity definition from the prediction types tabulated in a confusion matrix.

The total number of instances *N* and these three statistics fully characterise a unique confusion matrix and therefore fully describe the performance of a model against a test set. The prevalence and the total number of instances *N* characterise the test set, whereas sensitivity and specificity characterise the performance of the model.

**Fig. 2:**
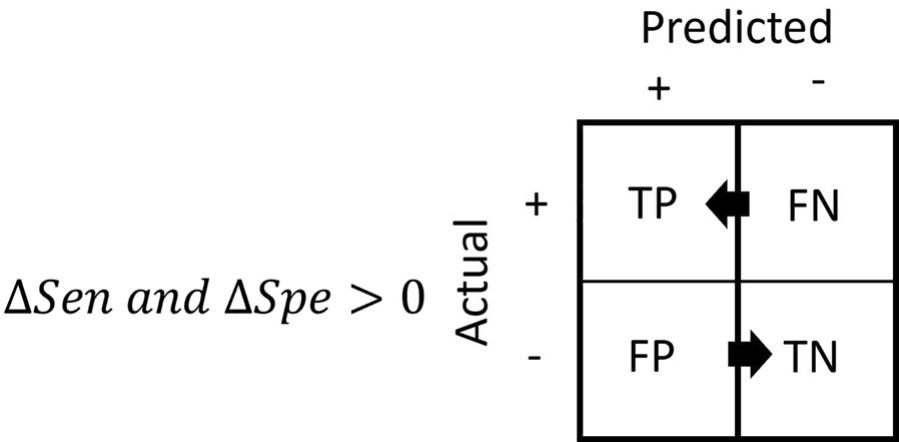
Model performance improves when the variation in sensitivity and specificity is greater than zero. Both positive variations indicate a relative increase in the count of true positive and true negative instances with a concomitant relative decrease in false positive and false negative

When a model is validated against more than one test set, the variation in the values of the sensitivity and the specificity allows the modeller to describe the relative performance of a model. Based on the variation in sensitivity and specificity values, there exist three broad descriptions of the model performance. One of them is subjective and use case specific. This scenario occurs when the variations in sensitivity and specificity change in opposite directions (e.g., the sensitivity increases while the specificity decreases). On the other hand, the two other scenarios are objective and occur when the variations in both sensitivity and specificity change in the same direction. When both variations are positive, or in other words, both sensitivity and specificity increase, one may say that the model performs better (Fig. 2). In contrast, when both variations are negative, the model performs worse.

**Fig. 3:**
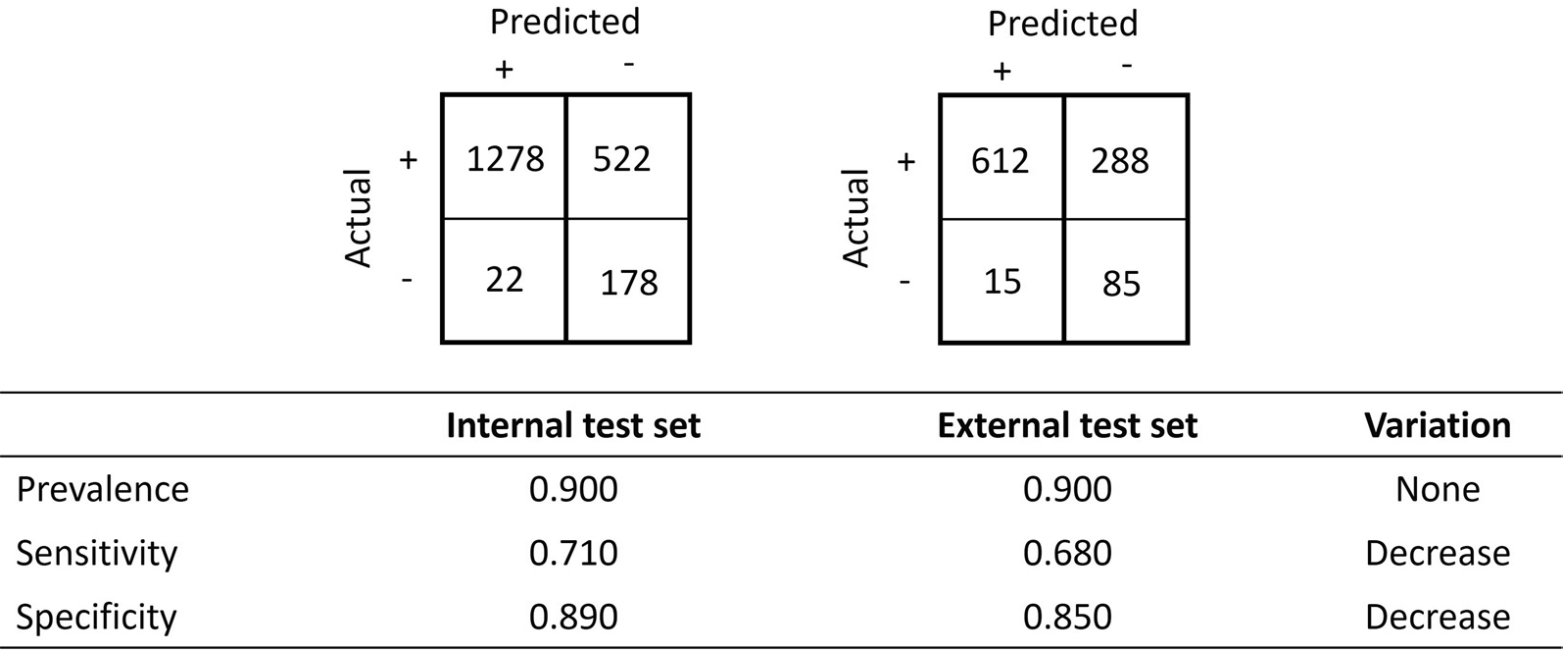
A model validated against an internal and external dataset of the same prevalence with their respective confusion matrix and their value of sensitivity, specificity, prevalence and their respective qualitative variation. Both the variation in sensitivity and specificity decreases; the model underperforms, as one would expect when exposed to the external dataset

This scenario is exemplified with synthetic data, as a model is validated against an external test set and is expected to underperform when compared to an internal test set. The synthetic data describe the performances of a model validated against an internal and external dataset of both positive prevalence 0.900 (i.e., 90.0% of instances have a positive class) and a total of instances of 2000 and 1000, respectively. For each dataset, the confusion matrix and the values of sensitivity, specificity, prevalence and their respective qualitative variation are reported in Fig. 3. Although both confusion matrices display good performance for the model under investigation, both sensitivity and specificity decreased, which indicates, with no ambiguity and as expected, that the model underperformed when exposed to the external dataset.

**Fig. 4:**
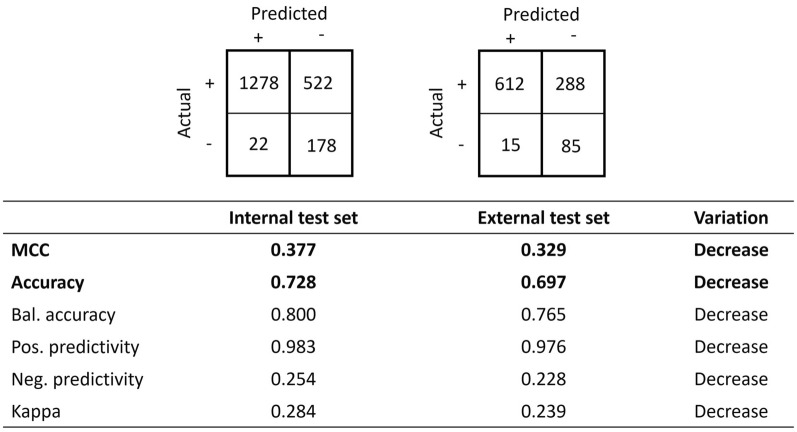
Values of the Matthews correlation coefficient, accuracy, balanced accuracy, positive predictivity, negative predictivity and Cohen’s kappa coefficient. The statistics that will be discussed in detail are in bold

Many other statistics may be defined and computed from a confusion matrix. Such statistics include accuracy, balanced accuracy, Matthews’ coefficient correlation, positive predictivity, negative predictivity, Cohen’s kappa coefficient, F1 score, informedness and markedness. The above example in Fig. 3 can be completed with the values of additional statistics, and as expected, all vary in the same direction. A decrease is observed for all the reported statistics, including accuracy and Matthew's coefficient correlation, as shown in Fig. 4. For the purpose of illustrating the main concepts, this manuscript will focus on accuracy and Matthews’ coefficient correlation, and we will later generalise the proposed principles to other metrics.

Examples of metrics benefiting from these principles are summarised in Additional file 3.

### The issue of comparing performance metrics under nonconstant prevalence

**Fig. 5:**
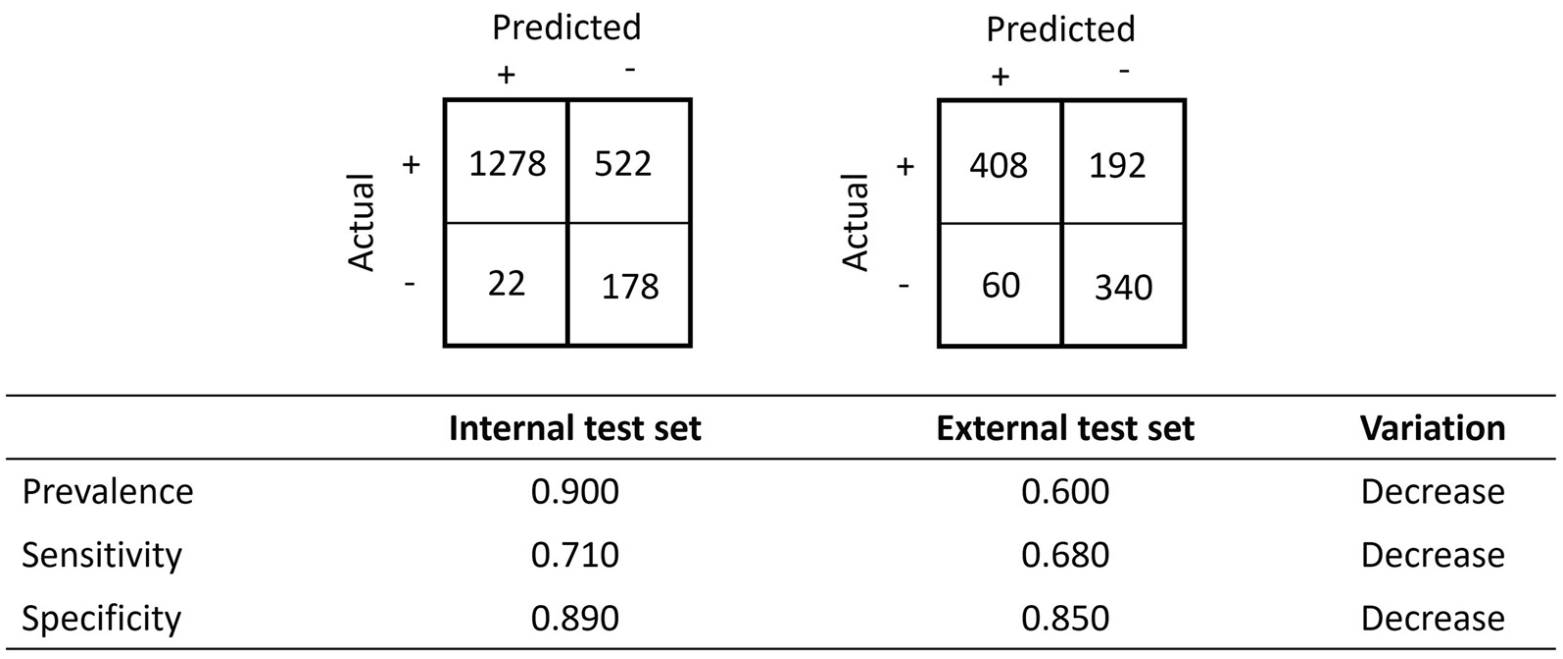
A model validated against an internal and external dataset of different prevalence with their respective confusion matrix and their value of sensitivity, specificity, prevalence and their respective qualitative variation. Both the variation in sensitivity and specificity decreases: the model underperforms as one would expect when exposed to the external dataset

In the previous example, the prevalences of the internal and external test sets were the same. When this statement is no longer true, unexpected results may arise and lead to incorrect conclusions. The synthetic data shown in Fig. 5 represent the data of a model that is validated with an external and an internal dataset with a total number of instances of 2000 and 1000 and a prevalence of 0.900 and 0.600, respectively. The corresponding external confusion matrix has changed, but the values of sensitivity and specificity remain identical. Therefore, one may again conclude that the model’s performance decreases when exposed to the external dataset.

**Fig. 6:**
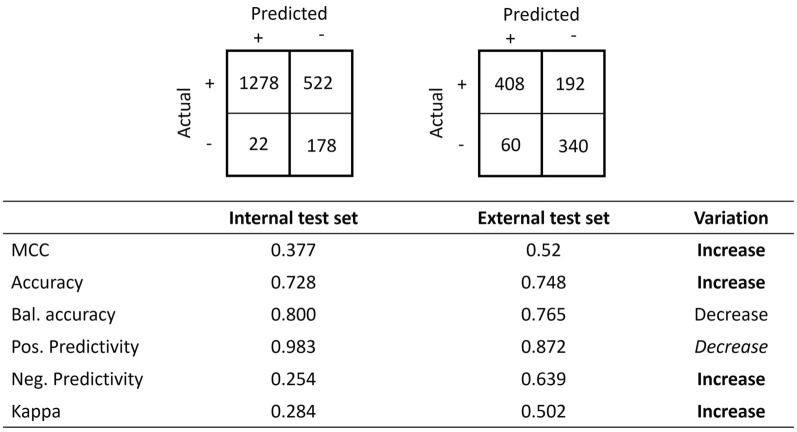
Values of the Matthews correlation coefficient, accuracy, balanced accuracy, positive predictivity, negative predictivity and Cohen’s kappa coefficient and their qualitative variation. Values in bold indicate an unexpected variation when compared to an increase in both sensitivity and specificity. Values in italics show an over- or underestimated quantitative variation

However, this time, when considering other performance metrics, we can observe that some metrics are increasing. For instance, metric values such as Matthews’ correlation coefficient, accuracy, positive predictivity, and Cohen’s kappa coefficient increase and therefore would inform the observer that the model performs better when validated against the external dataset, which feels counterintuitive given that both sensitivity and specificity decrease (Fig. 6).

Balanced accuracy variation, on the other hand, is consistent with the expected outcome, and as will be demonstrated later in this manuscript, balanced accuracy is the performance metric accuracy calibrated to a test set with a prevalence of 0.500 and therefore prevalence-independent.

The same principle of recalibrating the metric to a balanced prevalence can be generalised and applied to other metrics and is the topic of the following section.

## Towards balanced performance metrics

This section will derive a confusion matrix expressed with sensitivity, specificity, prevalence and the total number of instances *N* in a dataset in place of the four types of prediction: true positive, true negative, false positive and false negative. This derived confusion matrix may be used to express any performance metrics as a function of sensitivity, specificity and prevalence, and in this manuscript, we describe this translation in detail for accuracy and Matthews’ correlation coefficient. The derivation for other performance metrics is presented in Additional file 3.

### Derived confusion matrix

To turn a confusion matrix into a derived confusion matrix, the four types of prediction need to be expressed as functions of either sensitivity or specificity, prevalence and the total number of instances *N*.

Eq. 1 may be rearranged to give the count of positive and negative instances of a test set as a function of prevalence and *N*.

$$\begin{gathered} Pre = \frac{TP + FN}{N} \hfill \\ \Leftrightarrow TP + FN = Pre \cdot N \hfill\\ \end{gathered}$$

and

$$\begin{gathered} Pre = \frac{TP + FN}{N} \hfill\\ \Leftrightarrow 1 - Pre = 1 - \frac{TP + FN}{N} \hfill\\ = \frac{FP + TN}{N} \hfill\\ \Leftrightarrow TN + FP = (1 - Pre) \cdot N \hfill\\ \end{gathered}$$

Thus, the count of positive instances is the sum of true positive and false negative counts and is shown in Eq. (4).

4 $$TP+FN=Pre\cdot N$$

Equation 4. Count of positive instances as a function of prevalence and *N*.

The count of negative instances is the sum of the true negative and false positive counts and is shown in Eq. (5).

5 $$FP+TN=\left(1-Pre\right)\cdot N$$

Equation 5. Count of negative instances as a function of prevalence and *N*.

The expressions of Eqs. (4) and (5) appear in the denominator of Eq. (2) for sensitivity and Eq. (3) for specificity, respectively, where they can be substituted and rearranged to give an expression of the true positive and negative counts. Starting from Eq. (2) for sensitivity

$$\begin{gathered} Sen = \frac{TP}{{TP + FN}} = \frac{TP}{{Pre \cdot N}} \hfill \\ \Leftrightarrow TP = Sen \cdot Pre \cdot N \hfill \\ \end{gathered}$$

and starting from Eq. (3) for specificity

$$\begin{gathered} Spe = \frac{TP}{{TP + FN}} = \frac{TN}{{(1 - Pre) \cdot N}} \hfill \\ \Leftrightarrow TN = Spe \cdot (1 - Pre) \cdot N \hfill \\ \end{gathered}$$

give respectively the true positive count as a function of sensitivity, prevalence and *N* shown in Eq. (6) and the true negative count as a function of specificity, prevalence and *N* shown in Eq. (7).

6 $${\varvec{T}}{\varvec{P}}={\varvec{S}}{\varvec{e}}{\varvec{n}}\cdot {\varvec{P}}{\varvec{r}}{\varvec{e}}\cdot {\varvec{N}}$$

Equation 6. True positive count as a function of sensitivity, prevalence and *N*.

7 $${\varvec{T}}{\varvec{N}}={\varvec{S}}{\varvec{p}}{\varvec{e}}\cdot \left(1-{\varvec{P}}{\varvec{r}}{\varvec{e}}\right)\cdot {\varvec{N}}$$

Equation 7. True negative count as a function of specificity, prevalence and *N*.

Eq. (2) for sensitivity may be rearranged as follows:

$$\begin{gathered} Sen = \frac{TP}{{TP + FN}} \hfill \\ 1 - Sen = 1 - \frac{TP}{{TP + FN}} = \frac{FN}{{TP + FN}} \hfill \\ \Leftrightarrow TN = Spe \cdot (1 - Pre) \cdot N \hfill \\ \end{gathered}$$

where the denominator may be substituted by Eq. (4) and rearranged. Thus:

$$\begin{gathered} 1 - Sen = \frac{FN}{{TP + FN}} = \frac{FN}{{Pre \cdot N}} \hfill \\ \Leftrightarrow FN = (1 - Sen) \cdot Pre \cdot N \hfill \\ \end{gathered}$$

which gives the false negative count as a function of sensitivity, prevalence, and *N,* as shown in Eq. (8).

8 $${\varvec{F}}{\varvec{N}}=(1-{\varvec{S}}{\varvec{e}}{\varvec{n}})\cdot {\varvec{P}}{\varvec{r}}{\varvec{e}}\cdot {\varvec{N}}$$

Equation 8. False negative count as a function of sensitivity, prevalence and *N*.

In the same manner, Eq. (3) for specificity may be rearranged as follows:

$$\begin{gathered} Spe = \frac{TN}{{FP + TN}} \hfill \\ 1 - Spe = 1 - \frac{TN}{{FP + TN}} = \frac{FP}{{FP + TN}} \hfill \\ \end{gathered}$$

where the denominator may be substituted by Eq. (5) and rearranged. Thus:

$$\begin{gathered} 1 - Spe = 1 - \frac{FP}{{FP + TN}} = \frac{FP}{{(1 - Pre) \cdot N}} \hfill \\ \Leftrightarrow FP = (1 - Spe) \cdot (1 - Pre) \cdot N \hfill \\ \end{gathered}$$

which gives the false positive count as a function of specificity, prevalence and *N,* as shown in Eq. (9).

9 $${\varvec{F}}{\varvec{P}}=(1-{\varvec{S}}{\varvec{p}}{\varvec{e}})\cdot \left(1-{\varvec{P}}{\varvec{r}}{\varvec{e}}\right)\cdot {\varvec{N}}$$

Equation 9. False positive count as a function of specificity, prevalence and *N*.

**Fig. 7:**
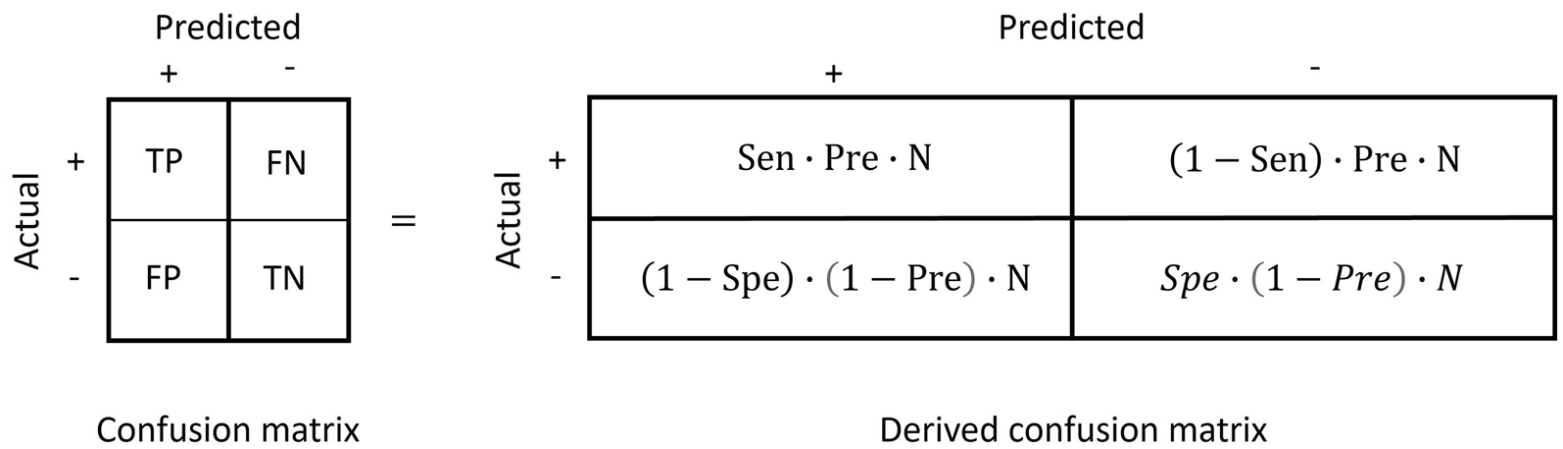
From the confusion matrix to the derived confusion matrix

Thus, the four prediction types of a confusion matrix can be substituted with Eqs. (6), (7), (8) and (9) to give the derived confusion matrix, as shown in Fig. 7.

The equivalence between a confusion matrix and a derived confusion matrix demonstrates two points. First, given that the performance of a model against a test set is fully described by its confusion matrix, it is also true for sensitivity, specificity, and prevalence. Second, from the derived confusion matrix, any metric that is defined as a function of the four types of prediction may also be expressed as a function of sensitivity, specificity and prevalence, where *N* cancels out to yield a metric that is normalised to a specific range. For example, sensitivity, specificity and accuracy range from 0 to 1, and Matthews’ correlation coefficient ranges from − 1 to 1.

### From accuracy to balanced accuracy

Accuracy is defined as the relative frequency of correct predictions relative to the total number of instances in the test set. The accuracy expression is shown in Eq. (10).

10 $$Acc=\frac{TP+TN}{N} \quad where \quad N=TP+FN+FP+TN$$

Equation 10. Accuracy definition from the prediction types tabulated in a confusion matrix.

The number of true positives and true negatives can be substituted with the corresponding expression of the derived confusion matrix and simplified to give accuracy as a function of sensitivity, specificity and prevalence, as shown in Eq. (11). The details of this derivation can be found in Additional file 3.

11 $${\mathbf{A}\mathbf{c}\mathbf{c}}^{\mathbf{P}\mathbf{r}\mathbf{e}}=\mathbf{S}\mathbf{e}\mathbf{n}\cdot \mathbf{P}\mathbf{r}\mathbf{e}+\mathbf{S}\mathbf{p}\mathbf{e}\cdot \left(1-\mathbf{P}\mathbf{r}\mathbf{e}\right)$$

Equation 11. Accuracy as a function of sensitivity, specificity and prevalence.

**Fig. 8:**
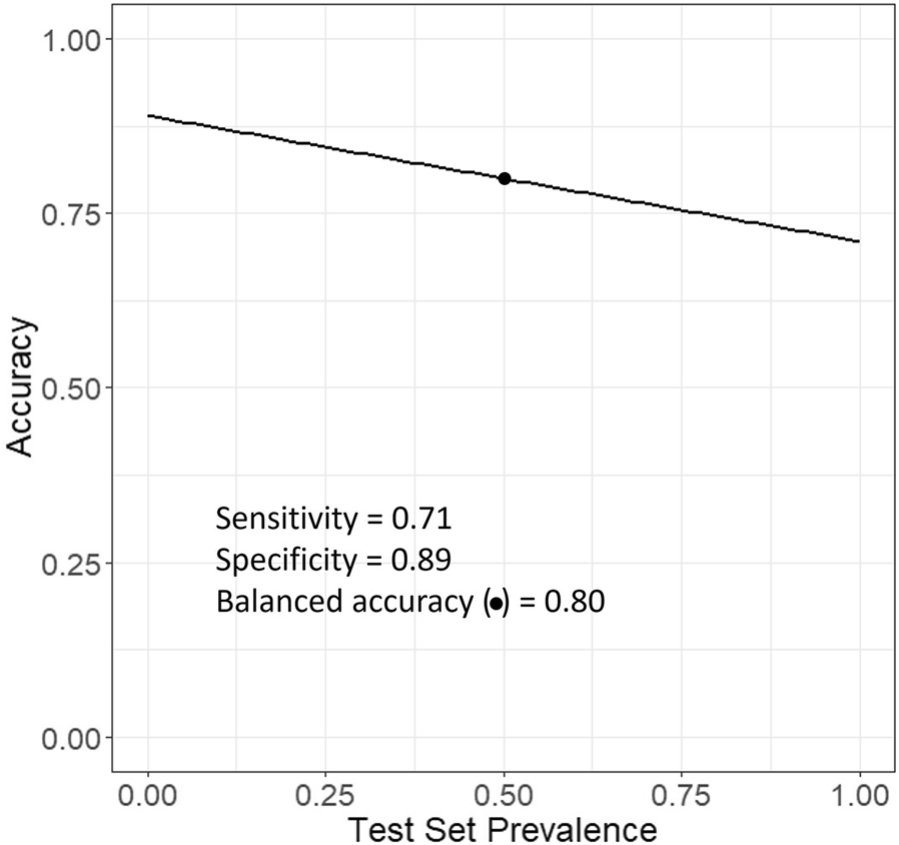
Linear relationship between accuracy and prevalence at constant values of sensitivity and specificity: 0.71 and 0.89, respectively. The dot marks the balanced accuracy

Equation (11) shows that accuracy is the average of sensitivity and specificity weighted by the prevalence and the negative prevalence, respectively, which is one minus the prevalence. At a prevalence of 0.500 (i.e., Pre = 0.500), Eq. (11) may be rewritten as follows:

$$\begin{aligned} Acc^{0.5} & = Sen \cdot \frac{1}{2} + Spe \cdot \left( {1 - \frac{1}{2}} \right) = Sen \cdot \frac{1}{2} + Spe \cdot \frac{1}{2} \hfill \\ & = \frac{Sen + Spe}{2} = Bal.Acc \hfill \\ \end{aligned}$$

and give the expression of balanced accuracy. Therefore, balanced accuracy is the performance metric accuracy calibrated to a test set with a positive prevalence of 50% (i.e., balanced test set). Consequently, the expression of accuracy in Eq. (11) allows the modeller to calibrate accuracy at a given prevalence. For example, Acc^0.9^ is the accuracy calibrated to a test set with a prevalence of 90%. The expression of accuracy in Eq. (11) allows the modeller to visualise how accuracy depends on prevalence to given values of sensitivity and specificity. Eq. (11) may be rearranged as shown below:

$$\begin{gathered} Acc^{Pre} = Sen \cdot Pre + Spe \cdot (1 - Pre) \hfill \\ \quad = Sen \cdot Pre + Spe - Spe \cdot Pre \hfill \\ \quad = (Sen - Spe) \cdot \Pr e + Spe \hfill \\ \end{gathered}$$

and give a linear relationship between accuracy and prevalence when the values of sensitivity and specificity are constant. In other words, this relationship gives the value of accuracy for prevalence ranging from 0 to 1 when sensitivity and specificity are fixed. Figure 8 illustrates this linear relationship when sensitivity and specificity equal 0.71 and 0.89, respectively. Additional files 2 and 4 provide R scripts to be used in RStudio and Additional file 1 is a JupyterLab notebook that allows the user to set values of sensitivity and specificity and visualise how it affects the value of accuracy.

Sensitivity minus specificity (i.e., Sen – Spe) is the slope, and specificity (i.e., Spe) is the value of accuracy when prevalence tends to zero. When the prevalence tends towards one, the accuracy tends towards sensitivity. When sensitivity and specificity tend to be similar, the slope tends to be zero, and the accuracy tends to be the same as the sensitivity and specificity. Therefore, accuracy becomes independent of the prevalence when sensitivity and specificity values tend to be the same, which would be represented by a horizontal line. In other words, the prevalence has no effect when sensitivity and specificity have the same value. In the same vein, when the values of sensitivity and specificity diverge, the slope increases. Consequently, accuracy may be over- or underestimated when the prevalence value tends to be high or low.

### Form Matthews’ correlation coefficient to balanced Matthew’s correlation coefficient.

Matthews’ correlation coefficient indicates the correlation that exists between the actual and predicted classes. A value of one or minus one indicates a perfect positive or negative correlation, respectively, whereas a value of zero indicates an absence of correlation. Matthews’ correlation coefficient expression as a function of the four prediction types is shown in Eq. (12).

12 $$MCC=\frac{TP\cdot TN-FP\cdot FN}{\sqrt{\left(TP+FP\right)\left(TP+FN\right)\left(TN+FP\right)\left(TN+FN\right)}}$$

Equation 12. Matthews’ correlation coefficient definition from the prediction types tabulated in a confusion matrix.

Like accuracy, Matthew’s correlation coefficient can be expressed as a function of sensitivity, specificity, and prevalence, as shown in Eq. (13). The details of this derivation can be found in the Additional file 3.

13 $${{\varvec{M}}{\varvec{C}}{\varvec{C}}}^{{\varvec{P}}{\varvec{r}}{\varvec{e}}}=\frac{{\varvec{S}}{\varvec{e}}{\varvec{n}}+{\varvec{S}}{\varvec{p}}{\varvec{e}}-1}{\sqrt{\left[{\varvec{S}}{\varvec{e}}{\varvec{n}}+\left(1-{\varvec{S}}{\varvec{p}}{\varvec{e}}\right)\frac{\left(1-{\varvec{P}}{\varvec{r}}{\varvec{e}}\right)}{{\varvec{P}}{\varvec{r}}{\varvec{e}}}\right]\left[{\varvec{S}}{\varvec{p}}{\varvec{e}}+\left(1-{\varvec{S}}{\varvec{e}}{\varvec{n}}\right)\frac{{\varvec{P}}{\varvec{r}}{\varvec{e}}}{\left(1-{\varvec{P}}{\varvec{r}}{\varvec{e}}\right)}\right]}}$$

Equation 13. Matthews’ correlation coefficient as a function of sensitivity, specificity and prevalence.

In the same manner as accuracy at a prevalence of 0.500 (i.e., Pre = 0.500), Eq. (13) may be rewritten to give the balanced Matthews’ correlation coefficient, as shown in Eq. (14). See Additional file 3 for the details of the proof.

14 $${{\varvec{M}}{\varvec{C}}{\varvec{C}}}^{0.5}=\frac{{\varvec{S}}{\varvec{e}}{\varvec{n}}+{\varvec{S}}{\varvec{p}}{\varvec{e}}-1}{\sqrt{1-{\left({\varvec{S}}{\varvec{e}}{\varvec{n}}-{\varvec{S}}{\varvec{p}}{\varvec{e}}\right)}^{2}}}={\varvec{B}}{\varvec{a}}{\varvec{l}}.\boldsymbol{ }{\varvec{M}}{\varvec{C}}{\varvec{C}}$$

Equation 14. Balanced Matthews’ correlation coefficient.

The numerator is the same expression as informedness, also known as Youden’s index [13]. The balanced accuracy values range from zero to one. However, this range may be scaled to minus one to one by applying a linear transformation on the expression of balanced accuracy. Given that the minimum and maximum values of balanced accuracy are 0 and 1, respectively, the linear transformation is given in the following expression and then applied to the definition of balanced accuracy: the average of sensitivity and specificity.

$${{\text{Bal}}.\mathrm{ Acc}}_{\left[-\mathrm{1,1}\right]}=2\cdot {\text{Bal}}. {{\text{Acc}}}_{\left[\mathrm{0,1}\right]}-1={\text{Sen}}+{\text{Spe}}-1$$

Thus, there are two noteworthy points to state. First, informedness and Youden’s coefficient are the same as balanced accuracy rescaled to the range of minus one to one. Second, the numerator of Matthews’ correlation coefficient in Eq. (13) and its balanced expression in Eq. (14) is also equivalent to balanced accuracy rescaled to the range of minus one. As such, the balanced Matthews’ coefficient correlation and the rescaled balanced accuracy differ only by the expression of the denominator of the latter and have the same value when sensitivity and specificity are equal. In that case, the denominator of the balanced Matthews’ coefficient correlation is evaluated to one.

$${\varvec{B}}{\varvec{a}}{\varvec{l}}.\boldsymbol{ }{\varvec{M}}{\varvec{C}}{\varvec{C}}=\boldsymbol{ }{{\varvec{B}}{\varvec{a}}{\varvec{l}}.\boldsymbol{ }{\varvec{A}}{\varvec{c}}{\varvec{c}}}_{\left[-1,1\right]} \quad \boldsymbol{ }{\varvec{w}}{\varvec{h}}{\varvec{e}}{\varvec{n}} \, \boldsymbol{ }{\varvec{S}}{\varvec{e}}{\varvec{n}}={\varvec{S}}{\varvec{p}}{\varvec{e}}$$

**Fig. 9:**
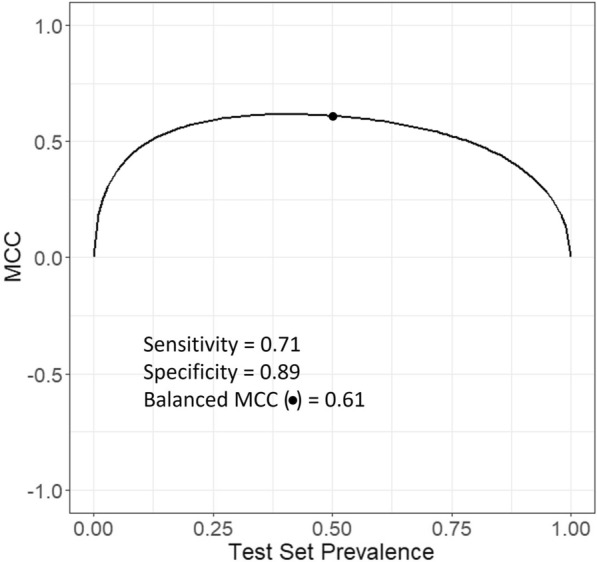
Nonlinear relationship between a Matthews’ correlation coefficient and prevalence at constant values of sensitivity and specificity: 0.71 and 0.89, respectively. The dot marks the balanced Matthews’ correlation coefficient

Eq. 13 shows a nonlinear relationship between Matthews’ correlation coefficient and prevalence at a given sensitivity and specificity. Fig. 9 illustrates this nonlinear relationship when the sensitivity and specificity are equal to 0.71 and 0.89, respectively. Additional files 2 and 4 provide R scripts to be used in RStudio and Additional file 1 is a JupyterLab notebook that allows the user to set values of sensitivity and specificity and visualise how it affects the value of Matthews’ correlation coefficient.

The curve has an umbrella-like shape. The umbrella-like shape may point upwards or downwards, which indicates a positive (MMC > 0) and negative (MCC < 0) correlation, respectively. This is due to the presence of two anchors, or two invariant points on the curve, at prevalence 0 and 1. A these two invariant points, the value of the Matthews’ correlation coefficient is zero regardless of the values of sensitivity and specificity, save when either or both are equal to 1 or 0 (see R scripts in RStudio in Additional files 2 and 4 or the JupyterLab notebook in Additional files 1). When sensitivity and specificity have different values, the umbrella-like shape is asymmetrical. It becomes symmetrical to the vertical line at a prevalence of 0.500 when sensitivity and specificity have the same value. In that specific case, the value of Matthews’ correlation coefficient reaches either its maximum or minimum value: the umbrella-like shape points upwards and downwards, respectively. As the values of sensitivity and specificity vary such as their sum tends to 1, the balanced accuracy rescaled between minus one and one (i.e., the numerator in Eq. (13) as demonstrated above) tends to zero, and the umbrella-like shape flattens. In contrast, as the balanced accuracy rescaled between minus one and one tends to 1 or − 1, the umbrella-like shape swells upwards or downwards, respectively. In contrast to accuracy, Matthews’ correlation coefficient does not have a linear relationship with prevalence at given values of sensitivity and specificity. However, its value remains dependent on prevalence. At extremely low or high values of prevalence, it drops sharply towards zero even though the values of sensitivity and specificity may be high.

## Discussion and use cases

Comparing performance metrics to assess a model during a validation when faced with test sets of nonconstant prevalence may lead to incorrect conclusions. The above example based on the synthetic data in Figs. 5 and 6 illustrates such an undesirable outcome. This issue arises because of the dependence of these metrics on prevalence and makes their comparison inappropriate as the prevalence shifts. Therefore, the notion of metrics that are independent of the prevalence of the dataset is needed. As we have seen, a solution to overcome this issue is to calibrate the metrics that need to be compared. A specific way of calibrating is balancing. As such, balanced accuracy, which is a well-known performance metric, satisfies the above notion. Therefore, values of balanced accuracy can be compared and will lead to a correct conclusion when evaluating a model under nonconstant prevalence. We have demonstrated the application of calibration and specifically balanced calibration to Matthews’ correlation coefficient metric and demonstrated how it mitigates the impact of prevalence. Additional file 3 describes the behaviour of positive predictivity, negative predictivity and Cohen’s kappa coefficient under nonconstant prevalence as a mathematical formula. Plots of these metrics as functions of sensitivity, specificity and prevalence as well as their balanced version as functions of sensitivity and specificity are also described. This manuscript also shows that the balanced accuracy is not a performance metric distinct from accuracy but in fact is an accuracy which is calibrated to a prevalence of 50%. Finally, rescaling the balanced accuracy to the range of − 1 to 1 showed that the resulting mathematical expression is the same as that of informedness or Youden’s index, which, in addition, happens to be the numerator of Matthews’ correlation and its balanced version.

When do one need to mind one’s prevalence? Prevalence needs to be accounted for whenever a shift occurs. Such a shift may occur in different use cases. Four uses cases are described in this discussion: validation based on the cluster splits methodology, nonstationary data streams, external versus internal validations, and the use of a framework of a domain of applicability of a model.

A cluster splits method may be used to generate test sets that cover different domains of the original test set. As a result, the prevalence of each test set may be different, and evaluating a model in these conditions with prevalence-dependent metrics may lead to incorrect conclusions.

These same inappropriate conditions may arise when there is a data stream, such as test sets with a temporal dependence. As time passes, the prevalence within the test window may vary and lead to undesirable consequences if the model is assessed with prevalence-dependent metrics.

**Fig. 10:**
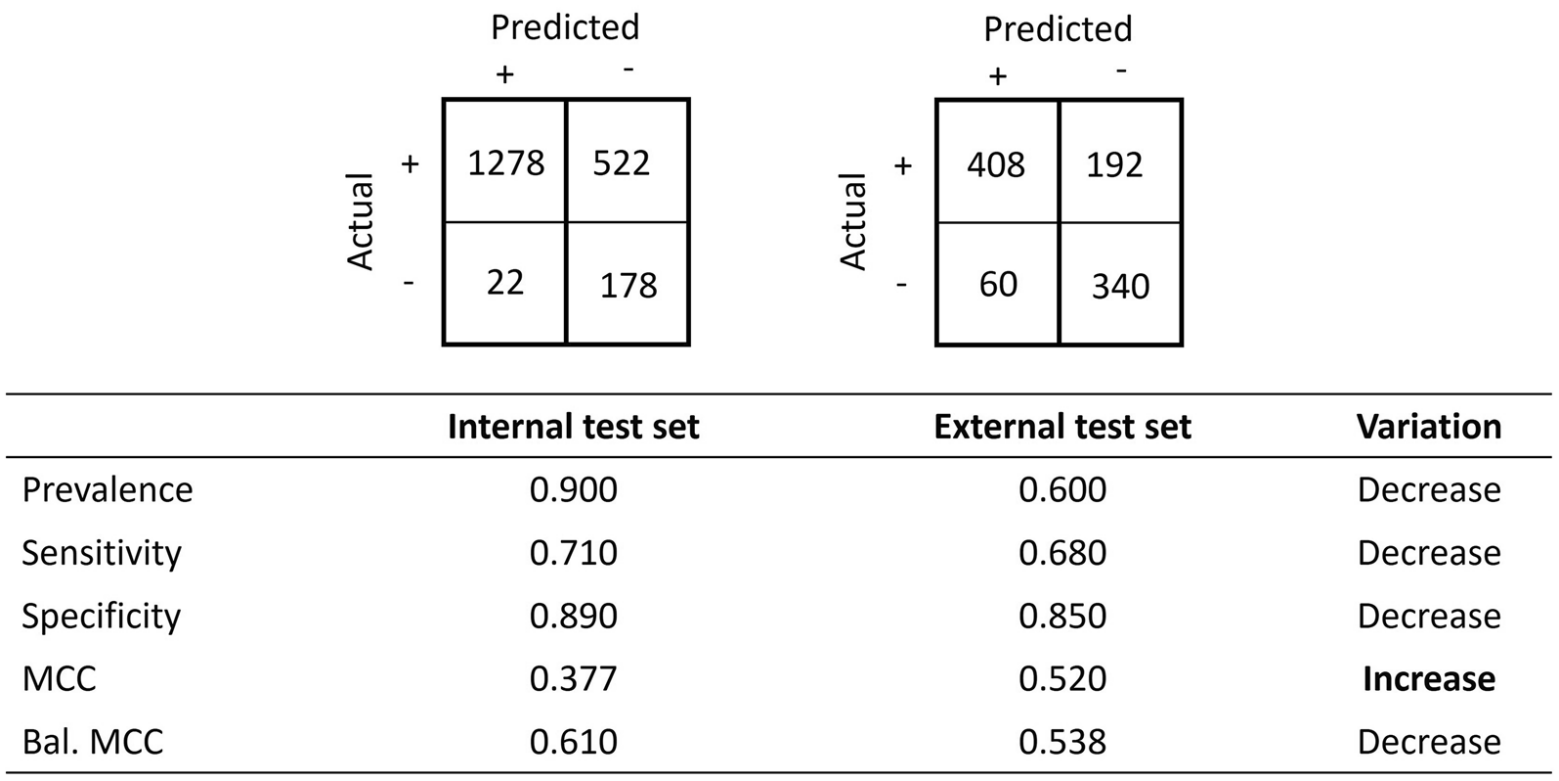
Values of Matthews’ correlation coefficient: balanced and not balanced in the internal versus external test set scenario. The variation of the latter agrees with those of sensitivity and specificity in contrast to that of the former

A model may perform well on an internal dataset. As such, it may be deployed and validated against external test sets where the model is expected to underperform while maintaining reasonable performances. However, there is no guarantee that the prevalence remains constant: the internal and external test sets may have different prevalence. Figures 5 and 6 illustrate such a scenario where the validation result seems to be counterintuitive. If accuracy or Matthews’ correlation coefficient were used as sole metrics, the model could be considered excellent when it is not the case at all. The use of balanced metrics corrects this misinterpretation and is shown in Fig. 10 with the same synthetic data as in Figs. 5 and 6 but showing the value of the balanced Matthews’ correlation coefficient. As expected, its value decreases in agreement with the variations observed for sensitivity and specificity.

**Fig. 11:**
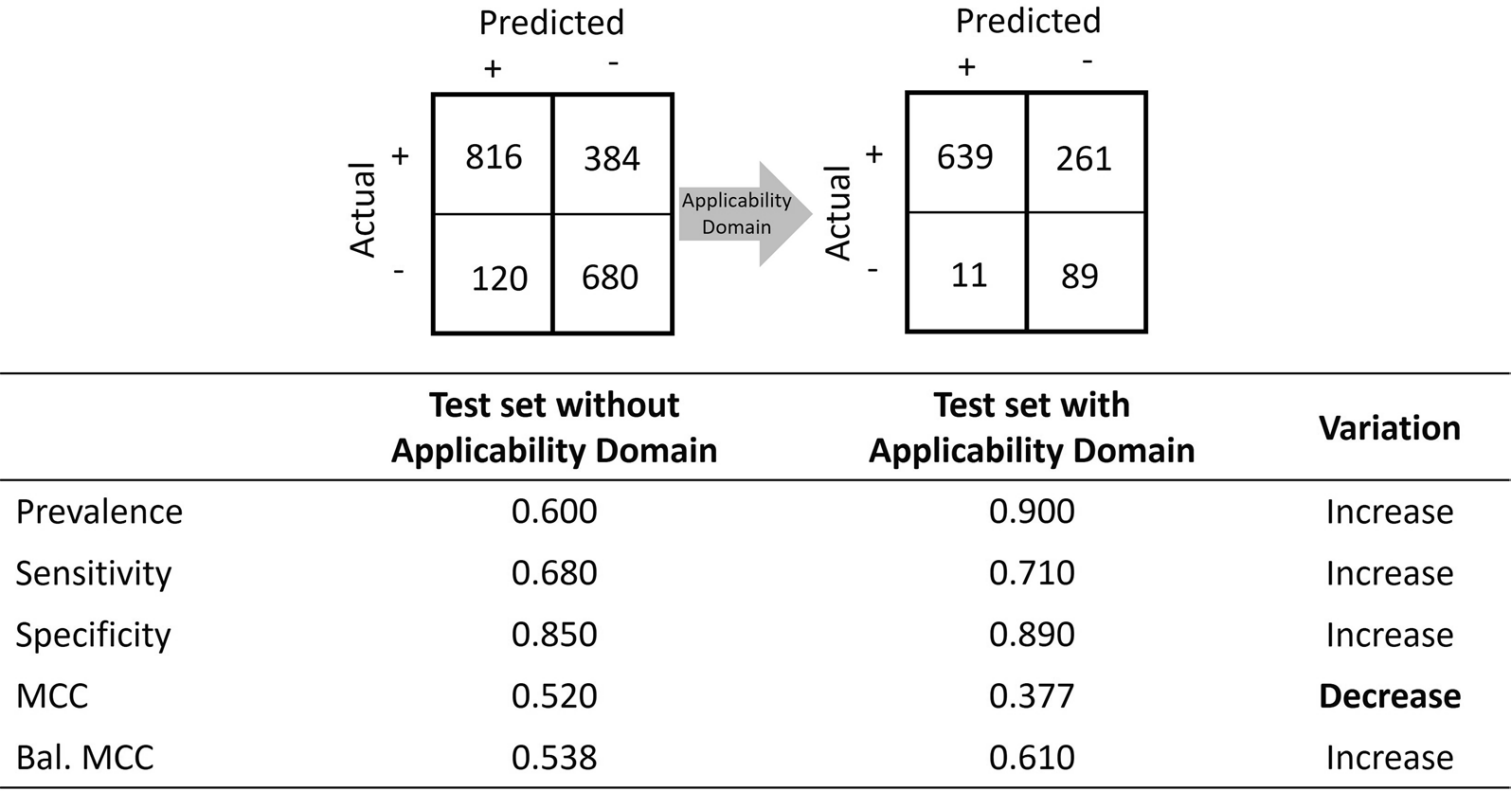
A tale of two Matthews’ correlation coefficients: balanced or not balanced?

A framework of domain of applicability may be applied to a test set before being validated. As such, the test set may contain a great deal of data out of domain, which are discarded as the model is deemed unfit to give meaningful predictions. In the presence of an applicability domain, the concept of coverage must be introduced. The coverage evaluates the proportion of instances for which the model can make a prediction. Therefore, the coverage of the model may decrease, but it is expected to perform better when meaningless predictions are removed from the test set. Hence, the size of the original test may be reduced in such a manner that the prevalence shifts. This use case is illustrated in Fig. 11 with synthetic data.

The initial dataset contains a total of 2000 instances with a prevalence of 0.600 when there is no applicability domain applied. The model under investigation shows good performance, with sensitivity and specificity values of 0.680 and 0.850, respectively. The Matthews’ correlation coefficient is also very satisfactory, with a value of 0.520. Nevertheless, sensitivity may be improved. A framework of the applicability domain may be used to achieve this goal with the knowledge that the coverage may drop. When applying the framework shown in Fig. 11, the coverage drops to 50%. Indeed, 1000 instances are out of domain and do not contribute to the calculation of the performance metrics. However, the sensitivity and specificity increased to 0.710 and 0.890, respectively, as expected. Paradoxically, Matthews’ correlation coefficient value drops to 0.377. If such a value is solely considered to assess the performance of the model, an incorrect conclusion is drawn. The reason comes from the fact that the prevalence of the instances in the applicability domain of the model increased to 0.900 from 0.600. A prevalence shift occurred. In this condition, Matthews’ correlation coefficients under nonconstant prevalence cannot be compared and may lead to incorrect conclusions. To correct this misleading result, a balanced/calibrated Matthews’ correlation coefficient may be used, which is illustrated with its balanced value in Fig. 11. The balanced Matthews’ correlation coefficients increase from 0.538 to 0.610, as expected, and lead to the proper conclusion.

## Conclusion

This manuscript lays out a simplified and harmonised approach to the issue of comparing prevalence dependant metrics under nonconstant prevalence experiments. This includes the introduction of the derived confusion matrix that provides a mathematical tool to express any metrics as a function of sensitivity, specificity and prevalence. In contrast to the literature, it also avoids the introduction of additional metrics, coefficients or frameworks to solve or describe the above issue. As a result, the concepts described in the literature are harmonised in this manuscript, which only uses the well-known metrics of sensitivity, specificity and prevalence. The derived confusion matrix also highlights that sensitivity, specificity and prevalence fully characterise the performance of a model. Based on this derived confusion matrix, we propose a general method to calibrate performance metrics with respect to the prevalence, and we recommend using a balanced prevalence of 0.5 as a reference calibration to compare model performances under nonconstant prevalence. For instance, the balanced accuracy is an accuracy that is calibrated to a prevalence of 50%. The same principle can be applied to any metric derived from the confusion matrix. We demonstrate the value of the approach on Matthews' correlation coefficient  leading to balanced Matthews' correlation coefficient, a robust global performance metric even under nonconstant prevalence. Finally, rescaling the balanced accuracy to the range of − 1 to 1 showed that the resulting mathematical expression is the same as that of informedness or Youden’s index. In addition, it appears to be in the numerator of Matthews’ correlation and its balanced version. This manuscript highlights the benefits of calibrated/balanced metrics for robust model evaluation and provides a formal grounding tailored to key use cases in cheminformatics research where prevalence fluctuations may occur. It is hoped that by increasing accessibility and demonstrating relevance to core cheminformatics applications, this work will lead to wider consideration of prevalence effects and increased uptake of calibrated/balanced metrics in future studies. Three final words: mind your prevalence!

### Supplementary Information

**Additional file 1.** A JupyterLab notebook. Upon execution, an interactive plot shows the relationship between performance metrics and prevalence at given values of sensitivity and specificity. The values of sensitivity and specificity may be modified with sliding bars. Check boxes allow the user to select what performance metrics to display.

**Additional file 2.** A R file that contains a R script that may be used in RStudio. It must source the Additional file 4, that needs to be renamed "UtilityFunctions.R" upon saving, so it can execute properly. Upon execution, an interactive plot shows the relationship between performance metrics and prevalence at given values of sensitivity and specificity. The values of sensitivity and specificity may be modified with sliding bars. Check boxes allow the user to select what performance metrics to display. The controls appears upon clicking on the cog icon in the top left corner of the Plots window of RStudio.

**Additional file 3.** A word document that describes in detail the derivation of the performance metrics accuracy, Matthews’ correlation coefficient, positive predictivity, negative predictivity and Cohen’s kappa coefficient as functions of sensitivity, specificity and prevalence as well as their balanced version. Each plot and their corresponding function show the relationship that exists between the performance metrics and the prevalence at given values of sensitivity and specificity.

**Additional file 4.** A R file that contains utily functions and must to be renamed "UtilityFunctions.R" upon saving for the proper execution of the R script in Additional file 2.

## Data Availability

Additional information and materials of this manuscript have been deposited in the GitHub repository MindYourPrevalence. The repository can be accessed at https://github.com/LhasaLimited/MindYourPrevalence.
