# Supplementary material for: Mind your prevalence!
Source: J Cheminform. 2024 Apr 15;16:43. doi: 10.1186/s13321-024-00837-w (PMC11017531; doi:10.1186/s13321-024-00837-w)
Supplement: Supplementary file 1 — Additional file 1. A JupyterLab notebook. Upon execution, an interactive plot shows the relationship between performance metrics and prevalence at given values of sensitivity and specificity. The values of sensitivity and specificity may be modified with sliding bars. Check boxes allow the user to select what performance metrics to display. [file 13321_2024_837_MOESM1_ESM.docx]

**Mind Your Prevalence!**

Sébastien J.J. Guesné*, Thierry Hanser, Stéphane Werner, Samuel Boobier, Shaylyn Scott

Lhasa Limited, Granary Wharf House, 2 Canal Wharf, Leeds, West Yorkshire, LS11 5PS, UK.

*Corresponding Author – Sébastien J. J. Guesné [sebastien.guesne@lhasalimited.org](mailto:sebastien.guesne@lhasalimited.org)

**Supplementary Information**

This supplementary information is intended to give the reader an understanding of the relationships that exist between performance metrics (e.g., accuracy, Matthew’s correlation coefficient) that are calculated from a binary confusion matrix with the sensitivity and specificity of a model and the class prevalence of the test set against which the model under investigation is validated. All these performance metrics can be derived as functions of sensitivity, specificity and class prevalence. The derivation starts from the derived confusion matrix, as shown in Figure 1.


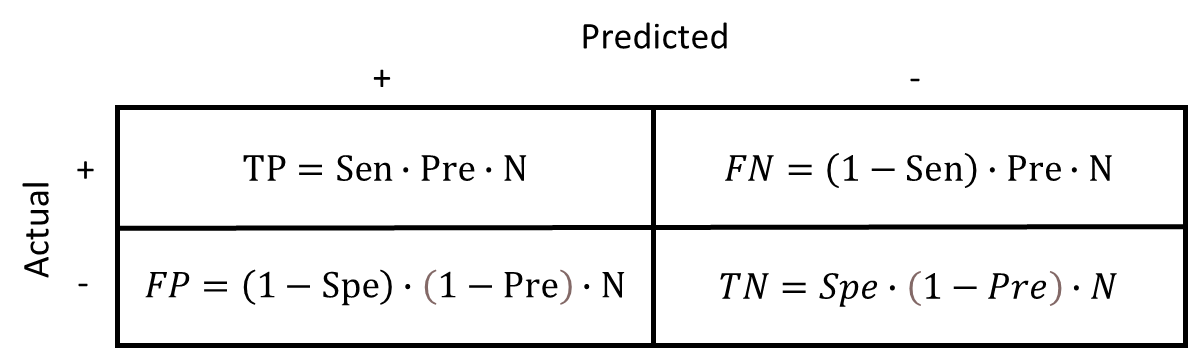


Figure 1. Derived confusion matrix.

These relationships or functions are presented as a plot of the value of performance metrics on the y-axis against the class prevalence on the x-axis at set values of sensitivity and specificity. The class prevalence ranges from 0 to 1 and represents the proportion of the selected class versus the rest of the classes in the case of multiclass classification problems. The confusion matrix of a multiclass classification problem is reduced to binary confusion matrices where the selected class is the class of interest and is defined as the positive class. The rest of the classes are defined as the negative class.

From these relationships, the notion of calibrated/balanced metrics is derived.

# Accuracy

## Derivation of accuracy as a function of sensitivity, specificity and prevalence

$$Acc.=\frac{TP+TN}{N}$$

$$=\frac{Pre\cdot Sen\cdot N+Spe\cdot\left( 1-Pre \right)\cdot N}{N}$$

$$Acc.= Pre\cdot Sen+Spe\cdot\left( 1-Pre \right)$$

$${\boldsymbol{Acc}\boldsymbol{.}}^{\boldsymbol{Pre}}\mathbf{=}\mathbf{Pre}\boldsymbol{\cdot}\mathbf{Sen}\mathbf{+}\mathbf{Spe}\boldsymbol{\cdot}\left( \mathbf{1}\mathbf{-}\mathbf{Pre} \right)$$

Equation 1. Accuracy.

## Linear relationship between accuracy and prevalence


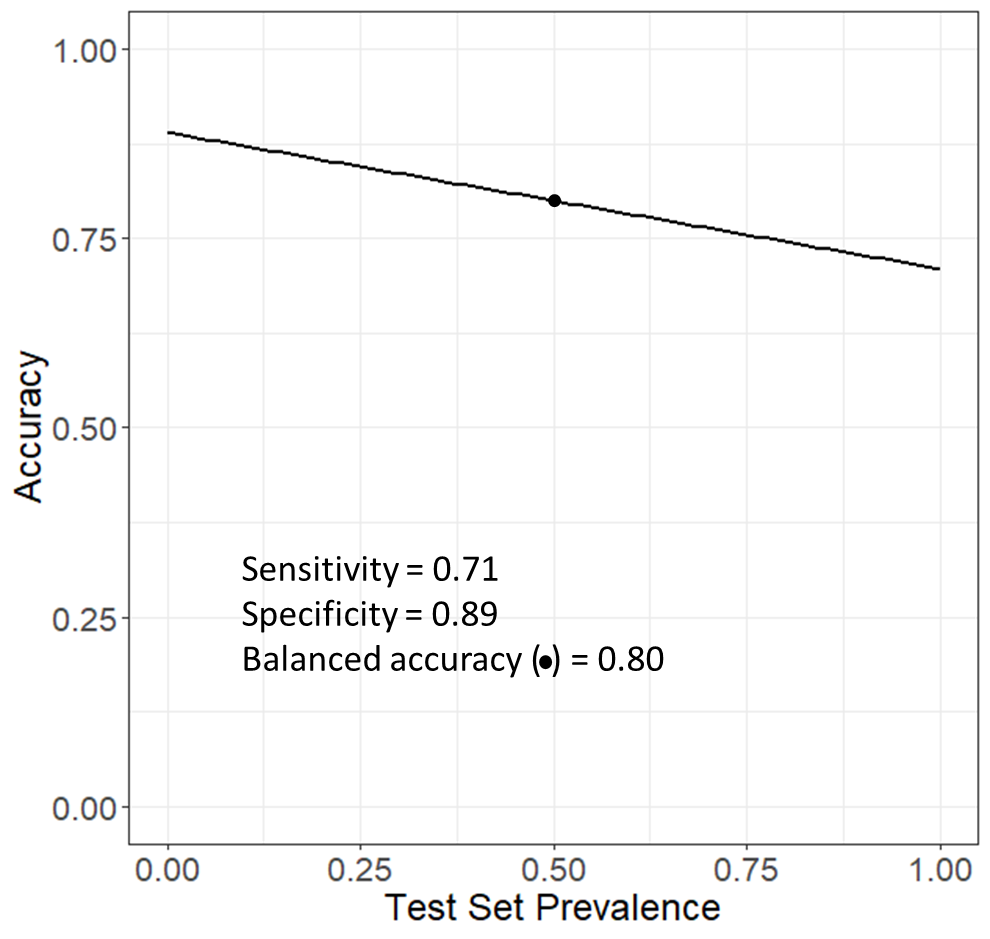


Figure 2. Accuracy versus test set prevalence for values of sensitivity and specificity of 0.71 and 0.89, respectively. The dot marks the balanced accuracy.

## Derivation of balanced accuracy

$${Acc.}^{0.5}=\frac{1}{2}\cdot Sen+\left( 1-\frac{1}{2} \right)\cdot\mathrm{Spe}$$

$$=\frac{1}{2}\cdot Sen+\frac{1}{2}\cdot\mathrm{Spe}$$

$$=\frac{Sen+Spe}{2} =Bal. Acc.$$

$$\mathbf{Bal. Acc.=}\frac{\mathbf{Sen+Spe}}{\mathbf{2}}$$

Equation 2. Balanced accuracy.

# Positive Predictivity

## Derivation of positive predictivity as a function of sensitivity, specificity and prevalence

$$Pos.Predictivity=\frac{\mathrm{TP}}{TP+FP}$$

$$=\frac{Pre\cdot N\cdot Sen}{Pre\cdot N\cdot Sen+\left( 1-Spe \right)\cdot\left( 1-Pre \right)\cdot N}$$

$$=\frac{Pre\cdot Sen}{N\cdot\left[ Pre\cdot Sen+\left( 1-Spe \right)\cdot\left( 1-Pre \right) \right]}$$

$$Pos.Predictivity=\frac{Pre\cdot Sen}{Pre\cdot Sen+\left( 1-Spe \right)\cdot\left( 1-Pre \right)}$$

$${\mathbf{Pos}\mathbf{.}\mathbf{Predictivity}}^{\mathbf{Pre}}\mathbf{=}\frac{\mathbf{Pre}\boldsymbol{\cdot}\mathbf{Sen}}{\mathbf{Pre}\boldsymbol{\cdot}\mathbf{Sen}\mathbf{+}\left( \mathbf{1}\mathbf{-}\mathbf{Spe} \right)\boldsymbol{\cdot}\left( \mathbf{1}\mathbf{-}\mathbf{Pre} \right)}$$

Equation 3. Positive predictivity.

## Nonlinear relationship between positive predictivity and prevalence


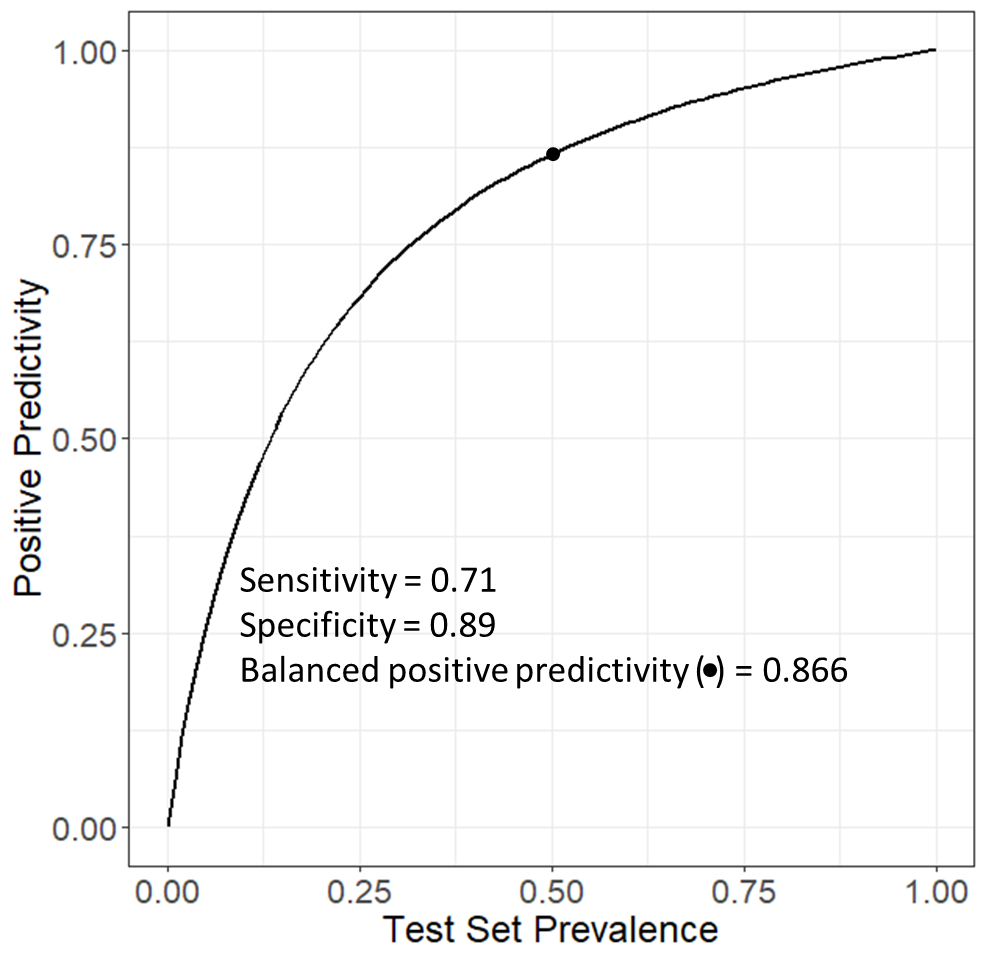


Figure 3. Positive predictivity versus test set prevalence for values of sensitivity and specificity of 0.71 and 0.89, respectively. The dot marks the balanced positive predictivity.

## Derivation of balanced positive predictivity

$${Pos. Predictivity}^{0.5}=\frac{\frac{1}{2}\cdot\mathrm{Sen}}{\frac{1}{2}\cdot Sen+\left( 1-Spe \right)\cdot\left( 1-\frac{1}{2} \right)}$$

$$=\frac{\frac{1}{2}\cdot\mathrm{Sen}}{\frac{1}{2}\cdot\left[ Sen+\left( 1-Spe \right) \right]}$$

$$=\frac{\mathrm{Sen}}{1+Sen-Spe}=Bal. Pos. Predictivity$$

$$\mathbf{Bal. Pos. Predictivity=}\frac{\mathbf{Sen}}{\mathbf{1+Sen-Spe}}$$

Equation 4. Balanced positive predictivity.

## Other expression of balanced positive predictivity in the literature

Landry *et al.*[1] and Trejo-Martin *et al.*[2] express the balanced positive predictivity (normalized positive predictivity therein) as a function of true positive, false positive and prevalence as shown in the following equation:

$$Normalised Pos. Predictivity=\frac{\mathrm{TP}/\mathrm{Pre}}{\mathrm{TP}/\mathrm{Pre}+\mathrm{FP}/\left( 1-Pre \right)}$$

When substituting TP and FP with their respective values from the derived confusion matrix, the normalized positive predictivity becomes the same expression as the balanced positive predictivity.

$$Normalised Pos. Predictivity=\frac{\mathrm{TP}/\mathrm{Pre}}{\mathrm{TP}/\mathrm{Pre}+\mathrm{FP}/\left( 1-Pre \right)}$$

$$= \frac{{Sen\cdot Pre\cdot N}/\mathrm{Pre}}{{Sen\cdot Pre\cdot N}/\mathrm{Pre}+{\left( 1-Spe \right)\cdot\left( 1-Pre \right)\cdot N}/\left( 1-Pre \right)}$$

$$=\frac{N}{N}\cdot\frac{{Sen\cdot Pre}/\mathrm{Pre}}{{Sen\cdot Pre}/\mathrm{Pre}+{\left( 1-Spe \right)\cdot\left( 1-Pre \right)}/\left( 1-Pre \right)}$$

$$=\frac{\mathrm{Sen}}{Sen+\left( 1-Spe \right)}$$

$$= \frac{\mathrm{Sen}}{1+Sen-Spe}= Bal. Pos. Predictivity$$

# Negative Predictivity

## Derivation of negative predictivity as a function of sensitivity, specificity and prevalence

$$Neg. Predictivity= \frac{\mathrm{TN}}{FN+TN}$$

$$=\frac{Spe\cdot\left( 1-Pre \right)\cdot N}{\left( 1-Sen \right)\cdot Pre\cdot N+Spe\cdot\left( 1-Pre \right)\cdot N}$$

$$=\frac{N\cdot Spe\cdot\left( 1-Pre \right)}{N\cdot\left[ \left( 1-Sen \right)\cdot Pre+Spe\cdot\left( 1-Pre \right) \right]}$$

$$Neg. Predictivity=\frac{Spe\cdot\left( 1-Pre \right)}{\left( 1-Sen \right)\cdot Pre+Spe\cdot\left( 1-Pre \right)}$$

$${\mathbf{Neg}\mathbf{.}\mathbf{Predictivity}}^{\boldsymbol{Pre}}\mathbf{=}\frac{\mathbf{Spe}\boldsymbol{\cdot}\left( \mathbf{1}\mathbf{-}\mathbf{Pre} \right)}{\left( \mathbf{1}\mathbf{-}\mathbf{Sen} \right)\boldsymbol{\cdot}\mathbf{Pre}\mathbf{+}\mathbf{Spe}\boldsymbol{\cdot}\left( \mathbf{1}\mathbf{-}\mathbf{Pre} \right)}$$

Equation 5. Negative predictivity.

## Nonlinear relationship between negative predictivity and prevalence


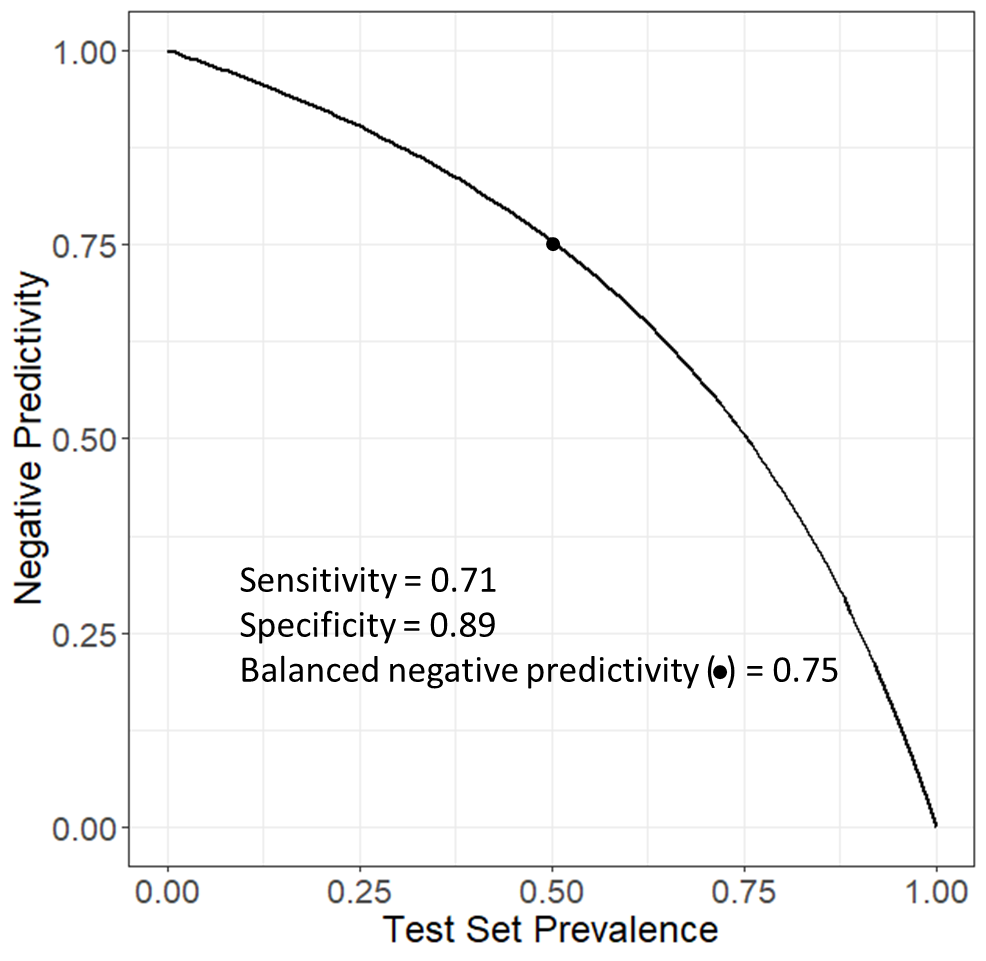


Figure 4. Negative predictivity versus test set prevalence for values of sensitivity and specificity of 0.71 and 0.89, respectively. The dot marks the balanced negative predictivity.

## Derivation of balanced negative predictivity

$${Neg. Predictivity}^{0.5}=\frac{\frac{1}{2}\cdot\mathrm{Spe}}{\frac{1}{2}\cdot Spe+\left( 1-Sen \right)\cdot\left( 1-\frac{1}{2} \right)}$$

$$=\frac{\frac{1}{2}\cdot\mathrm{Spe}}{\frac{1}{2}\cdot\left[ Spe+\left( 1-Sen \right) \right]}$$

$$=\frac{\mathrm{Spe}}{1-Sen+Spe}= Bal. Neg. Predictivity$$

$$\mathbf{Bal. Neg. Predictivity=}\frac{\mathbf{Spe}}{\mathbf{1-Sen+Spe}}$$

Equation 6. Balanced negative predictivity.

## Other expression of balanced negative predictivity in the literature

Landry *et al*[1] and Trejo-Martin *et al.*[2] express the balanced negative predictivity (normalized negative predictivity therein) as a function of true negative, false negative and prevalence as shown in the following equation:

$$Normalised Neg. Predictivity=\frac{\mathrm{TN}/\left( 1-Pre \right)}{\mathrm{TN}/\left( 1-Pre \right)+\mathrm{FN}/\mathrm{Pre}}$$

When substituting TN and FN with their respective values from the derived confusion matrix, the normalized negative predictivity becomes the same expression as the balanced negative predictivity.

$$Normalised Neg. Predictivity=\frac{\mathrm{TN}/\left( 1-Pre \right)}{\mathrm{TN}/\left( 1-Pre \right)+\mathrm{FN}/\mathrm{Pre}}$$

$$= \frac{{Spe\cdot\left( 1-Pre \right)\cdot N}/\left( 1-Pre \right)}{{Spe\cdot\left( 1-Pre \right)\cdot N}/\left( 1-Pre \right)+{\left( 1-Sen \right)\cdot Pre\cdot N}/\mathrm{Pre}}$$

$$=\frac{N}{N}\cdot\frac{{Spe\cdot\left( 1-Pre \right)}/\left( 1-Pre \right)}{{Spe\cdot\left( 1-Pre \right)}/\left( 1-Pre \right)+{\left( 1-Sen \right)\cdot Pre}/\mathrm{Pre}}$$

$$=\frac{\mathrm{Spe}}{Spe+\left( 1-Sen \right)}$$

$$=\frac{\mathrm{Spe}}{1-Sen+Spe}= Bal. Neg. Predictivity$$

# Matthews’ correlation coefficient

## Derivation of Matthews’ correlation coefficient as a function of sensitivity, specificity and prevalence

$$MCC=\frac{TP\cdot TN-FP\cdot FN}{\sqrt{\left( TP+FP \right)\left( TP+FN \right)\left( TN+FP \right)\left( TN+FN \right)}}=\frac{A}{\sqrt{B}}$$

$$A=TP\cdot TN-FP\cdot FN$$

$$=N^{2}\cdot\left( 1-Pre \right)\cdot Pre\cdot\left[ Sen\cdot\mathrm{Spe}-\left( 1-Spe \right)\cdot\left( 1-Sen \right) \right]$$

$$A=N^{2}\cdot\left( 1-Pre \right)\cdot Pre\cdot\left( Sen+Spe-1 \right)$$

$$B=\left( TP+FP \right)\left( TP+FN \right)\left( TN+FP \right)\left( TN+FN \right)$$

$$TP+FP=Pre\cdot N\cdot Sen+\left( 1-Spe \right)\cdot\left( 1-Pre \right)\cdot N$$

$$=N\cdot Pre\cdot Sen+\left( 1-Spe \right)\cdot\left( 1-Pre \right)\cdot N$$

$$=N\cdot Pre\cdot\left[ Sen+\left( 1-Spe \right)\cdot\frac{\left( 1-Pre \right)}{\mathrm{Pre}} \right]$$

$$TP+FN=Sen\cdot Pre\cdot N+\left( 1-Sen \right)\cdot Pre\cdot N$$

$$=Sen\cdot Pre\cdot N+Pre\cdot N-Sen\cdot Pre\cdot N$$

$$=N\cdot Pre$$

$$TN+FP=Spe\cdot\left( 1-Pre \right)\cdot N+\left( 1-Spe \right)\cdot\left( 1-Pre \right)\cdot N$$

$$=\left( 1-Pre \right)\cdot N\cdot\left[ Spe+\left( 1-Spe \right) \right]$$

$$=\left( 1-Pre \right)\cdot N\cdot\left( Spe+1-Spe \right)$$

$$=\left( 1-Pre \right)\cdot N$$

$$TN+FN=\left( 1-Sen \right)\cdot Pre\cdot N+Spe\cdot\left( 1-Pre \right)\cdot N$$

$$=N\cdot\left[ \left( 1-Sen \right)\cdot Pre+Spe\cdot\left( 1-Pre \right) \right]$$

$$= \left( 1-Pre \right)\cdot N\cdot\left[ Spe+\left( 1-Sen \right)\cdot\frac{\mathrm{Pre}}{\left( 1-Pre \right)} \right]$$

$$B=\left( TP+FP \right)\left( TP+FN \right)\left( TN+FP \right)\left( TN+FN \right)$$

$$=N^{4}\cdot\mathrm{Pre}^{2}\cdot\left( 1-Pre \right)^{2}\cdot\left[ Sen+\left( 1-Spe \right)\cdot\frac{\left( 1-Pre \right)}{\mathrm{Pre}} \right]\cdot\left[ Spe+\left( 1-Sen \right)\cdot\frac{\mathrm{Pre}}{\left( 1-Pre \right)} \right]$$

$$\sqrt{B}=N^{2}\cdot Pre\cdot\left( 1-Pre \right)\cdot\sqrt{\left[ Sen+\left( 1-Spe \right)\cdot\frac{\left( 1-Pre \right)}{\mathrm{Pre}} \right]\cdot\left[ Spe+\left( 1-Sen \right)\cdot\frac{\mathrm{Pre}}{\left( 1-Pre \right)} \right]}$$

$$\mathrm{MCC}^{\mathrm{Pre}}=\frac{A}{\sqrt{B}}=\frac{Sen+Spe-1}{\sqrt{\left[ Sen+\left( 1-Spe \right)\cdot\frac{\left( 1-Pre \right)}{\mathrm{Pre}} \right]\cdot\left[ Spe+\left( 1-Sen \right)\cdot\frac{\mathrm{Pre}}{\left( 1-Pre \right)} \right]}}$$

$$\mathbf{MCC}^{\mathbf{Pre}}\mathbf{=}\frac{\mathbf{Sen}\mathbf{+}\mathbf{Spe}\mathbf{-}\mathbf{1}}{\sqrt{\left[ \mathbf{Sen}\mathbf{+}\left( \mathbf{1}\mathbf{-}\mathbf{Spe} \right)\boldsymbol{\cdot}\frac{\left( \mathbf{1}\mathbf{-}\mathbf{Pre} \right)}{\mathbf{Pre}} \right]\boldsymbol{\cdot}\left[ \mathbf{Spe}\mathbf{+}\left( \mathbf{1}\mathbf{-}\mathbf{Sen} \right)\boldsymbol{\cdot}\frac{\mathbf{Pre}}{\left( \mathbf{1}\mathbf{-}\mathbf{Pre} \right)} \right]}}$$

Equation 7. Matthews’ correlation coefficient.

## Nonlinear relationship between Matthews’ correlation coefficient and prevalence


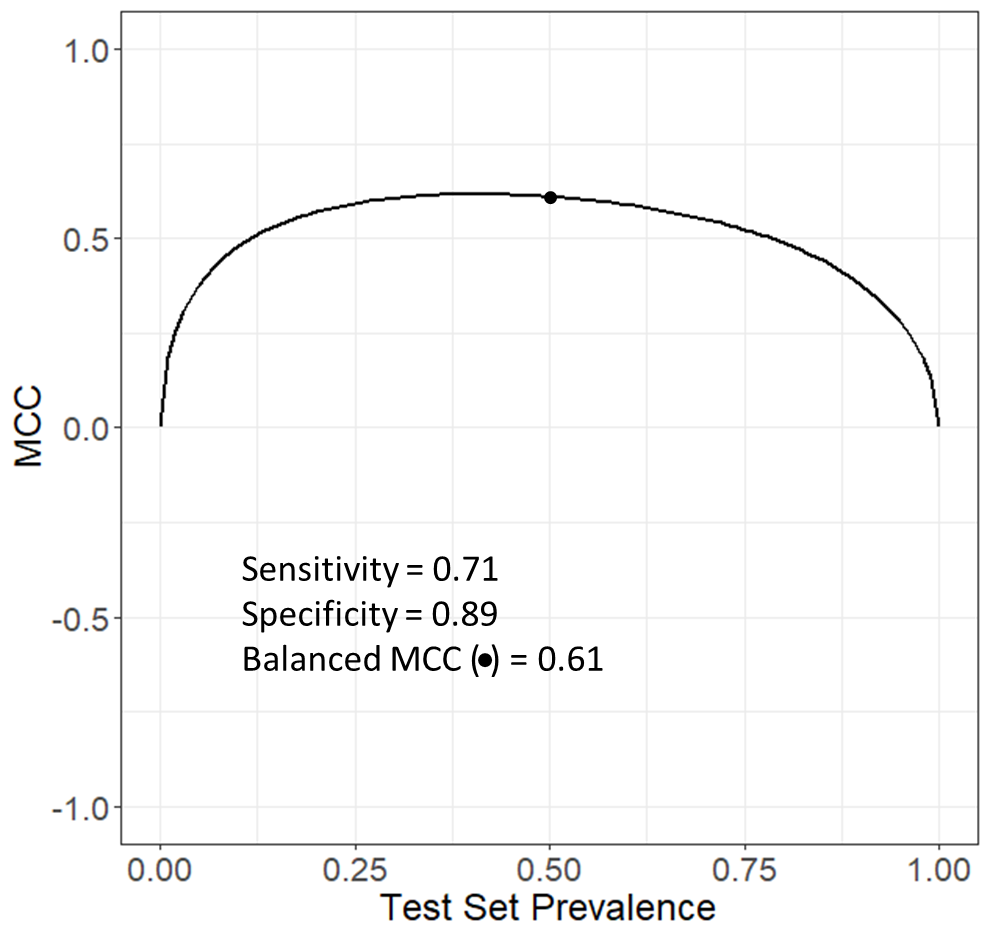


Figure 5. Matthews’ correlation coefficient versus test set prevalence for values of sensitivity and specificity of 0.71 and 0.89, respectively. The dot marks the balanced Matthews’ correlation coefficient.

## Derivation of balanced Matthews’ correlation coefficient

$$\mathrm{MCC}^{0.5}=\frac{Sen+Spe-1}{\sqrt{\left[ Sen+\left( 1-Spe \right)\cdot\frac{\left( 1-\frac{1}{2} \right)}{\frac{1}{2}} \right]\cdot\left[ Spe+\left( 1-Sen \right)\cdot\frac{\frac{1}{2}}{\left( 1-\frac{1}{2} \right)} \right]}}$$

$$=\frac{Sen+Spe-1}{\sqrt{\left[ Sen+\left( 1-Spe \right) \right]\cdot\left[ Spe+\left( 1-Sen \right) \right]}}$$

$$=\frac{Sen+Spe-1}{\sqrt{\left( Sen+1-Spe \right)\cdot\left( Spe+ 1-Sen \right)}}$$

$$=\frac{Sen+Spe-1}{\sqrt{2\cdot Spe\cdot Sen-\mathrm{Spe}^{2}-\mathrm{Sen}^{2}+1}}$$

$$=\frac{Sen+Spe-1}{\sqrt{1-\left( \mathrm{Spe}^{2}-2\cdot Spe\cdot Sen+\mathrm{Sen}^{2} \right)}}$$

$$=\frac{Sen+Spe-1}{\sqrt{1-\left( Sen-Spe \right)^{2}}}=Bal. MCC$$

$$\mathbf{Bal. MCC=}\frac{\mathbf{Sen+Spe-1}}{\sqrt{\mathbf{1-}\left( \mathbf{Sen-Spe} \right)^{\mathbf{2}}}}$$

Equation 8. Balanced Matthews’ correlation coefficient.

# Cohen’s kappa coefficient

## Derivation of Cohen’s kappa coefficient as a function of sensitivity, specificity and prevalence

$$Kappa=\frac{2\cdot\left( TP\cdot TN-FP\cdot FN \right)}{\left( TP+FP \right)\left( TN+FP \right)+\left( TP+FN \right)\left( TN+FN \right)}=\frac{2\cdot A}{C}$$

See Matthews’ correlation coefficient for the expression of A.

$$C=\left( TP+FP \right)\left( TN+FP \right)+\left( TP+FN \right)\left( TN+FN \right)$$

$$=N^{2}\cdot Pre\cdot\left( 1-Pre \right)\left\{ \left[ Sen+\left( 1-Spe \right)\cdot\frac{\left( 1-Pre \right)}{\mathrm{Pre}} \right]+\left[ Spe+\left( 1-Sen \right)\cdot\frac{\mathrm{Pre}}{\left( 1-Pre \right)} \right] \right\}$$

$$\mathrm{Kappa}^{\mathrm{Pre}}=\frac{2\cdot A}{C}=\frac{2\cdot\left( Sen+Spe-1 \right)}{\left[ Sen+\left( 1-Spe \right)\cdot\frac{\left( 1-Pre \right)}{\mathrm{Pre}} \right]+\left[ Spe+\left( 1-Sen \right)\cdot\frac{\mathrm{Pre}}{\left( 1-Pre \right)} \right]}$$

$$\mathbf{Kappa}^{\mathbf{Pre}}\mathbf{=}\frac{\mathbf{2}\boldsymbol{\cdot}\left( \mathbf{Sen}\mathbf{+}\mathbf{Spe}\mathbf{-}\mathbf{1} \right)}{\left[ \mathbf{Sen}\mathbf{+}\left( \mathbf{1}\mathbf{-}\mathbf{Spe} \right)\boldsymbol{\cdot}\frac{\left( \mathbf{1}\mathbf{-}\mathbf{Pre} \right)}{\mathbf{Pre}} \right]\mathbf{+}\left[ \mathbf{Spe}\mathbf{+}\left( \mathbf{1}\mathbf{-}\mathbf{Sen} \right)\boldsymbol{\cdot}\frac{\mathbf{Pre}}{\left( \mathbf{1}\mathbf{-}\mathbf{Pre} \right)} \right]}$$

Equation 9. Cohen’s kappa coefficient.

## Nonlinear relationship between Cohen’s kappa coefficient and prevalence


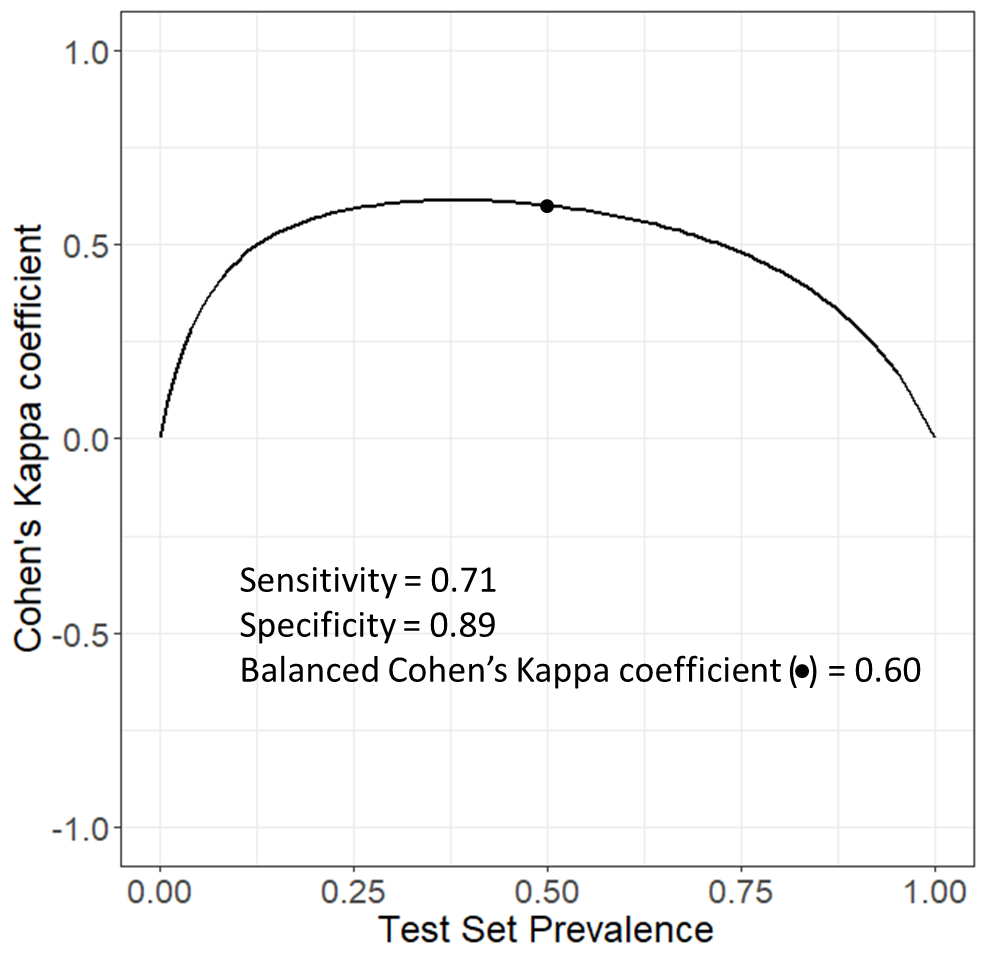


Figure 6. Cohen’s kappa coefficient versus test set prevalence for values of sensitivity and specificity of 0.71 and 0.89, respectively. The dot marks the balanced Cohen’s kappa coefficient.

## Derivation of balanced Cohen’s kappa coefficient

$$\mathrm{Kappa}^{0.5}=\frac{2\cdot\left( Sen+Spe-1 \right)}{\left[ Sen+\left( 1-Spe \right)\cdot\frac{\left( 1-\frac{1}{2} \right)}{\frac{1}{2}} \right]+\left[ Spe+\left( 1-Sen \right)\cdot\frac{\frac{1}{2}}{\left( 1-\frac{1}{2} \right)} \right]}$$

$$=\frac{2\cdot\left( Sen+Spe-1 \right)}{\left[ Sen+\left( 1-Spe \right) \right]+\left[ Spe+\left( 1-Sen \right) \right]}$$

$$=\frac{2\cdot\left( Sen+Spe-1 \right)}{Sen+1-Spe+Spe+1-Sen}$$

$$=\frac{2\cdot\left( Sen+Spe-1 \right)}{2}$$

$$=Sen+Spe-1= Bal. Kappa$$

$$\mathbf{Bal. Kappa= Sen+Spe-1}$$

Equation 10. Balanced Cohen’s kappa coefficient.

The numerator of Cohen’s kappa coefficients and the expression of its balanced version is the balanced accuracy rescaled to the range of -1 to 1, also known as informedness or Youden’s index.

# References

1. Landry C, Kim MT, Kruhlak NL, *et al* (2019) Transitioning to composite bacterial mutagenicity models in ICH M7 (Q)SAR analyses. Regul Toxicol Pharmacol 109:104488. https://doi.org/10.1016/j.yrtph.2019.104488

2. Trejo-Martin A, Bercu JP, Thresher A, *et al* (2022) Use of the bacterial reverse mutation assay to predict carcinogenicity of N-nitrosamines. Regul Toxicol Pharmacol 135:105247. https://doi.org/10.1016/j.yrtph.2022.105247
